# The Elements of Vital Statistics

**Published:** 1900-03

**Authors:** 


**The Elements of Vital Statistics.** By ARTHUR NEWSHOLME, M.D. Third Edition. Pp. xii., 353. London: Swan Sonnenschein & Co., Lim. 1899.

Since the appearance of Dr. Newsholme's *Vital Statistics* in 1889 the manual has become indispensable to all engaged in public health work. The new edition bears evidence of considerable labour bestowed on the revision of every section, and the information has been carefully modernised. Several new diagrams add to the value of the work, which appears in a new and improved form.

### Notes on Preparations for the Sick.

